# Quantifying conjugation rates in clinical and environmental matrices: a systematic review to inform risk assessment

**DOI:** 10.3389/frmbi.2024.1490240

**Published:** 2025-01-17

**Authors:** Hunter Quon, Lucia Ramirez, Blakeley Bagwell, Jennifer Moralez, Richard J. Sheppard, Allison J. Lopatkin, Kerry A. Hamilton

**Affiliations:** ^1^ School of Sustainable Engineering and the Built Environment, Arizona State University, Tempe, AZ, United States; ^2^ The Biodesign Center for Environmental Health Engineering, Arizona State University, Tempe, AZ, United States; ^3^ Department of Biology, Barnard College, New York, NY, United States; ^4^ Medical Research Council (MRC) Centre for Global Infectious Disease Analysis & World Health Organization (WHO) Collaborating Centre for Infectious Disease Modelling, Jameel Institute, School of Public Health, Imperial College London, London, United Kingdom; ^5^ Department of Chemical Engineering, University of Rochester, Rochester, NY, United States; ^6^ Department of Microbiology and Immunology, University of Rochester, Rochester, NY, United States

**Keywords:** horizontal gene transfer, conjugation, antimicrobial resistance, risk assessment, wastewater

## Abstract

**Introduction:**

Antimicrobial resistance (AMR) has become a major public health concern and challenge. The transfer of antimicrobial resistance genes (ARG) between bacteria and the movement of antibiotic resistant bacteria (ARB) between human, environmental, and animal reservoirs allows AMR to spread and drive its persistence. Modeling efforts are useful for providing understanding of fate and transport, dynamics, or probabilistic risk, but lack estimates of bacterial conjugation parameters to be used within these frameworks.

**Methods:**

A systematic literature review was conducted to summarize measured rates of conjugation for AMR and other resistances across a variety of settings, experimental media, and donor sources. Results: Across the 113 studies, reported conjugation frequencies and rates were examined in environmental, clinical, and animal/agricultural settings. The findings spanned over 12 orders of magnitude. From all studies, a subset of 25 were able to be analyzed for time-dependent rate estimation, which is most useful in modeling approaches. The highest rates were found in samples originating from wastewater sources or transferred in wastewater matrices, pointing to the significance and role of anthropogenic impacts on the environment in dissemination of AMR.

**Discussion:**

The results allowed us to identify knowledge gaps in measuring conjugation rates in key environmental exposure areas, such as biofilms, and in reporting experimental outputs for understanding cell growth and conjugation dynamics, such as donor, recipient and transconjugant densities over time.

## 1 Introduction

Over 2.8 million antibiotic resistant infections occur annually in the United States (US), and over 35,000 people die each year from these infections (CDC, 2019). Antibiotic resistant infections are estimated to cost the US over $2 billion annually (Thorpe et al., 2018). While overuse of antibiotics partly drives antimicrobial resistance (AMR), other environmental factors also contribute to the spread of antibiotic resistant bacteria (ARB), antibiotic resistance genes (ARG), and other mobile genetic elements (MGE) (Bengtsson-Palme et al., 2018; Berendonk et al., 2015; Larsson et al., 2018). Sewage and wastewater environments such as municipal wastewater, reclaimed or recycled wastewater, and hospital or pharmaceutical wastewaters have been highlighted as potential areas for focus. These areas are noted due to their contribution as environmental “hot spots” of AMR where ARG, ARB, antibiotics, heavy metals, pharmaceuticals, disinfectants, nutrients, and other stressors can co-mingle (Hong et al., 2018; Vikesland et al., 2017). Some outbreaks of ARB have been noted for originating from human exposure to water matrices (Gordon et al., 2017; Hayward, 2020) and epidemiological linkages observed between exposure to water environments and the threat of development of waterborne AMR diseases and enteric infections (Berendes et al., 2019; Chatterjee et al., 2018; Coleman et al., 2013).

**Figure 1:**
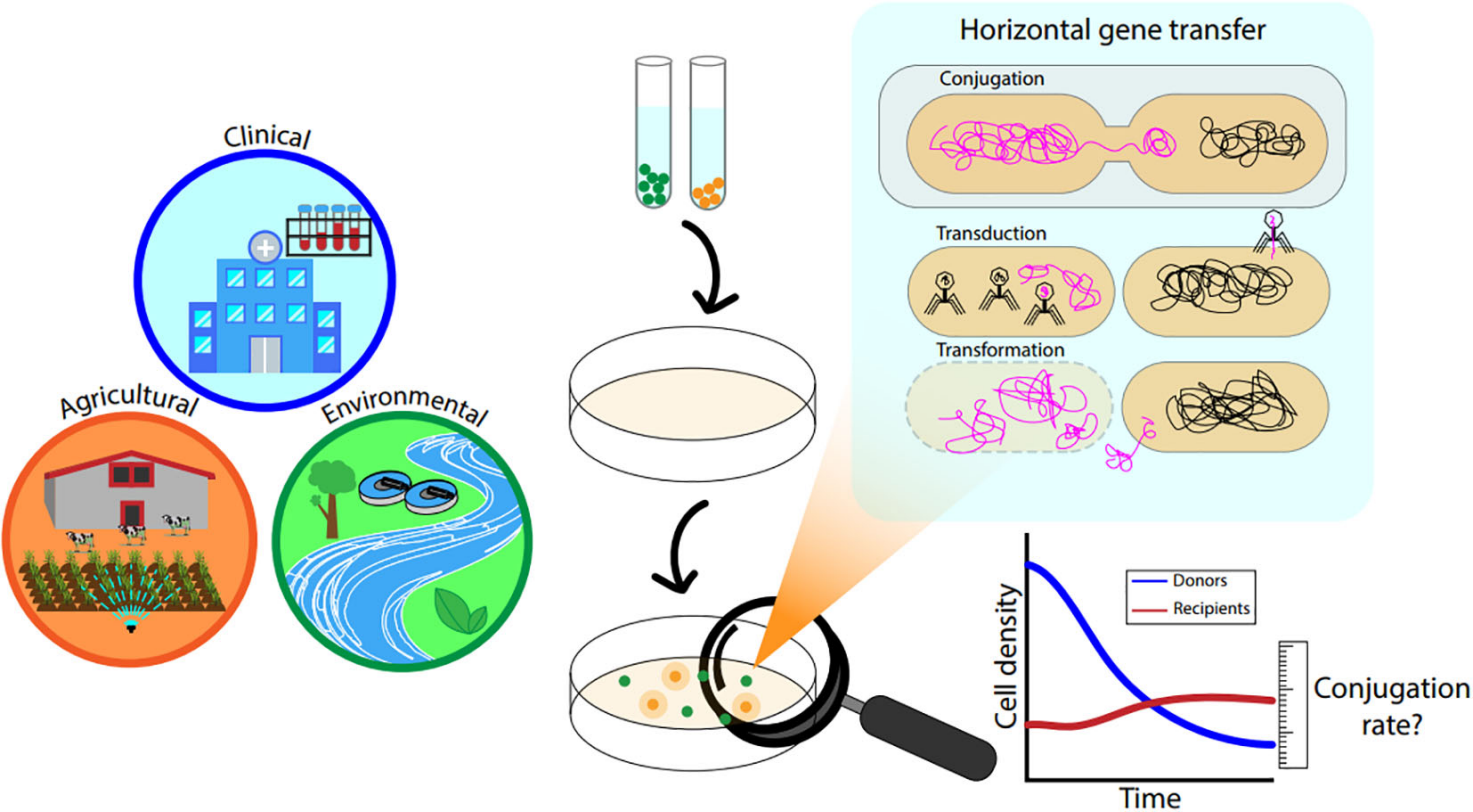
General graphic describing clinical and environmental interfaces related to HGT and HGT mechanisms with highlight on conjugation.

AMR can develop due to genetic mutations, recombination coupled with clonal expansion, or horizontal gene transfer (HGT) (Banerji et al., 2019; Boolchandani et al., 2019) whereby functional ARGs are directly shared amongst distinct cells, including those of different microbial species. ARGs can encode for processes associated with antibiotic resistance phenotypes, most commonly acting by altered drug transport, antibiotic target modification, or antibiotic degradation enzymes (Blair et al., 2015; Vikesland et al., 2017). HGT is thought to be a dominant process in the development and spread of AMR, and encompasses mechanisms of conjugation, transduction, and transformation (von Wintersdorff et al., 2016). The relative importance of plasmid conjugation, which is the process of transferring plasmids between a donor and recipient bacteria through direct contact (mating) (Griffiths et al., 2000), is emphasized for environmental matrices and AMR, since its efficiency is greater compared to other HGT mechanisms (von Wintersdorff et al., 2016), and the high prevalence of plasmids that often encode one or more ARGs (Pinilla-Redondo et al., 2018). The rate at which conjugation occurs is a function of multiple factors including host, recipient, and plasmid identities, as well as cell density, media type and environmental conditions (Pruden et al., 2018; Tamanai-Shacoori et al., 1995) (**Figure 1**).

Several modeling methods have been applied for predicting the rate of conjugation and its relevance to downstream AMR processes (Moralez et al., 2021), typically using frameworks of infectious disease modeling (Knight et al., 2018), evolutionary biology (Townsend et al., 2012), and quantitative microbial risk assessment (QMRA) (Njage and Buys, 2017, 2015). These studies have highlighted the need for information to parameterize kinetic conjugation models in both the human body and environment. Numerous authors have highlighted the need for accounting for HGT and particularly conjugation dynamics for assessing the risks of AMR in the water and wastewater context (Amarasiri et al., 2019; Banerji et al., 2019; Bengtsson-Palme et al., 2017; Bengtsson-Palme and Heb, 2019; Berendonk et al., 2015; Bürgmann et al., 2018; Gwenzi et al., 2020; Holmes et al., 2016; Hong et al., 2018; Knight et al., 2018; McEwen and Collignon, 2018; Rice et al., 2020; Rittmann et al., 1990; Smets et al., 1990). Other hotspots should be considered for their spread to the environment such as hospital wastewater and clinical settings (Harris et al., 2014; Lerminiaux and Cameron, 2019; Samreen et al., 2021), and animal or agricultural areas and wastes (Jadeja and Worrich, 2022; Topp et al., 2018).

Other reviews have analyzed rates of conjugation (Alderliesten et al., 2020; Ashelford et al., 2006; Hunter et al., 2008; Sheppard et al., 2020; Sorensen et al., 2005), and have noted that most studies have occurred at *in vitro* scales and/or in pure culture rather than in environmental or full-scale systems. The conjugation “rate” is often reported as a frequency, or ratio of quantities of transconjugants (T), donors (D), recipients (R), and/or plasmids (e.g. T/D, T/R) (Lopatkin et al., 2016b). Dynamics of conjugation, including rates of plasmid transfer or loss, are useful for quantification within biological processes such as wastewater treatment (Rittmann et al., 1990; Smets et al., 1990). However, the common reporting of conjugation frequencies often excludes metrics of time, cell density, or cell metabolism, thus adding difficulty in utilizing the reported conjugation results for modeling treatment, fate, or transport (Lopatkin et al., 2016b, 2017). One key area where this is important and yet to be explored is in QMRA models. A review of context, media, sources, and conjugation rates is necessary for understanding and applying HGT to such models and assessments.

There is a need to understand mechanistic interactions between microorganisms that play a role in the acquisition of AMR from an environmental source (e.g. sewage, clinical settings, agriculture, etc.). Improved knowledge of these determinants will allow for prioritization and assessment of AMR monitoring opportunities as well as management interventions to prevent the spread of AMR. In particular, knowledge gaps for measuring and assessing environmental sources and pathways (such as surface waters or sewage) of AMR continue to be areas of targeted research (Pruden et al., 2018). Therefore, our objectives for the current work are to: (1) systematically review the literature for quantitative conjugation frequencies or rates in different environmental or clinical settings; (2) summarize and compare the findings and measurements across key metrics and conditions; (3) provide recommendations for reporting experimental conjugation rates to best progress modeling efforts; and (4) identify gaps and suggestions for future experiments.

## 2 Methods

### 2.1 Data extraction and analysis

A systematic literature review was performed based on preferred reporting items for systematic reviews and meta-analysis (PRISMA) guidelines (Moher et al., 2015) to identify studies of HGT reported for environmental and clinical settings. Further details and a description of the inclusion criteria are in the **Supplementary Material**.

A single reviewer extracted data for each topic (water/sewage, clinical, and animal/agriculture respectively) from relevant papers and a second reviewer verified information from 10% of entries for each. Fields recorded included (1) experimental media; (2) origin of donor species/strain/plasmid; (3) identity of donor species/strain/plasmid; (4) origin of recipient species/strain/plasmid; (5) identity of recipient species/strain/plasmid; (6) type of antibiotic used to assess resistance; (7) genetic material transferred; (8) initial cell density; (9) experimental duration; (10) horizontal gene transfer rate information and applicable statistics and replicates reported; (11) units of horizontal gene transfer rate; and (12) quantification methods used (e.g. cell culture, microscopy, or quantitative polymerase chain reaction [qPCR]). Studies that met inclusion criteria had quantitative rates or rates presented in a graphical format that could be extracted were recorded in an Excel spreadsheet. Where data were only available in graphical format, data were extracted using Digitize It^©^ (Alcasa, 2016) data extraction software. Data were analyzed using summary statistics, statistical tests, and boxplots in R v.4.0.4 (2021).

### 2.2 Conjugation rate analysis for comparison

Generally, the densities of donor, recipient, and transconjugant cells at the end of experiments are used to report horizontal gene transfer or conjugation as a ratio or frequency based on transconjugant concentration over either donor or recipient concentration (T/D, T/R). Not all studies reported all fields listed above, nor did they report the concentrations of T, D, or R throughout the experiments. To analyze conjugation as a rate, that is, a time-dependent transfer of plasmids that results in a change in transconjugant population, we applied equations based on growth and plasmid transfer (Simonsen et al., 1990) as follows:


(1) $$\psi =\;\frac{N-N_{0}}{t-t_{0}}$$

(2) $$\gamma =\psi \ln\left(1+\frac{TN}{DR}\right)\frac{1}{N-N_{0}}$$

Where T, D, R, N are the concentrations of transconjugants, donors, recipients, and total cell density at the endpoint time *t*, resulting in a final rate of conjugation γ in units of ml cell^-1^ h^-1^. All reviewed studies were further analyzed for available data for rate conversion. Where cell densities or times were not reported, the following equation was used as a proxy for Equations 1 and Equations 2 as demonstrated by others (Sheppard et al., 2020; Zhong et al., 2012):


(3) $$\gamma^{*}=\;\frac{T}{DR}$$

## 3 Results

A total of 113 studies were analyzed for data extraction. The studies were categorically organized based on either their experimental matrix or the origin of donor/recipient species for analysis. After review, the chosen categories were environmental, clinical, and, due to some studies including *in vivo* experiments and unique environments and sources, animal/agricultural. 71 studies met the inclusion criteria for environmental (namely water such as rivers or wastewater) matrices, 42 studies met inclusion criteria in clinical studies (sourced from clinical isolates or conducted in laboratories without environmental source or matrix), and 16 were designated as animal/agricultural (sourced from agricultural settings or based on *in-vivo* experiments for animals) (**Supplementary Table S1**). Several studies could be classified as multi-category; for example, a strain originated from a clinical sample, but the conjugation experiments took place in a water matrix (Ohlsen et al., 2003). In other cases, both donor and recipient originated from similar matrices, for example, strains were isolated from a water environment and conjugation experiments took place in a water environment (Geisenberger et al., 1999), or donor and recipient strains were isolated from a water environment but conjugation experiments took place in another media (Fernandez-Astorga et al., 1992).

Across all studies, *E. coli* was the most prominent donor (39/71 environmental, 19/42 clinical, and 8/16 agricultural) and recipient species (42/71, 19/42, and 10/16). For environmental studies, other common donors were *Pseudomonas* spp. (18/71), *Enterobacter* spp. (5/71), and *Citrobacter* spp. (5/71). Remaining donors such as *Salmonella* spp., *Enterococcus* spp., and *Staphylococcus* spp., were used in three or fewer studies. The same trends were observed for recipients (*Pseudomonas* spp. 16/71, and *Enterococcus* spp. 5/71). Most of the donors were gram negative bacteria (64/71 environmental, 32/42 clinical, 13/16 agricultural) and similar for recipients (63/71 environmental, 28/42 clinical, 13/16 agricultural). With some overlap due to multiple measured bacteria, the remaining were gram positive donors (7/71 environmental, 12/42 clinical, 5/16 agricultural) and recipients (9/71 environmental, 16/42 clinical, 5/16 agricultural).

The resistance type transferred was assessed most for ampicillin (18/70 environmental, 5/45 clinical, 3/10 agricultural), kanamycin (20/70, 10/45, 0/10), tetracycline (31/45, 8/70, 4/10), and trimethoprim (11/70, 2/45, 1/10). Other lesser common resistances transferred were cefotaxime, colistin, sulfonamide, and gentamicin. Transferred resistances to metals were also measured in some studies, namely mercury (6/70 environmental), nickel (3/70), and copper, zinc, and cadmium (2/70 each).

Most commonly, conjugation results were described as frequencies or ratios of T/R (62/113) and T/D (51/113). Other units, such as T/ml or T/total cells were utilized in few studies (5/113) (**Supplementary Figure S1**). In addition to different experimental conditions and rates of transfer, the experimental time was widely variable across all studies (20 minutes to 31 days). Therefore, the conjugation across all unit types also covered a wide range, spanning over 12 orders of magnitude (**Supplementary Figure S1**). The majority of studies used culture-based methods with or without antibiotics in the media (85/113), or PCR (19/113) with the remaining quantifying cell counts using epifluorescence microscopy (6/113) or flow cytometry (3/13). From the 113 studies, 25 were analyzed for rate estimation with Equation 1-Equation 3. This was due to their reporting of ml cell^-1^ h^-1^ or data able to be extracted for the endpoint method. Only 25 of the 113 were able to be analyzed with this method, as many of the studies: 1) only reported final conjugation frequencies (T/R, T/D); 2) did not report cell counts of N, T, D, and/or R as they varied between time points (Equation 1, Equation 2); or Equation 3) did not report T, D, and R for use with Equation 3.This subgroup included 14 environmental, 12 clinical, and 6 animal/agricultural studies.

### 3.1 Conjugation frequencies

#### 3.1.1 Environmental frequencies

As the largest category (n=71/113), environmental studies in this review focused on bacteria isolated from water (such as rivers or seawater), wastewater, or soil/sediments (marine or riverbeds). Studies where conjugation rates of clinical isolates or laboratory collection samples were measured in an environmental medium (such as a wastewater matrix) were also categorized as environmental (**Supplementary Table S1**). Wastewater or activated sludge were utilized as an experimental medium for 11 studies and were a common source of donor bacteria (21/70) and/or recipient bacteria (15/70). Other water media were surface waters such as rivers, lakes, or canals (8 studies), seawater (7 studies), and stormwater (1 study). Non-water environmental media were river or marine sediments (2 studies), and reactors (6 studies). Agar, broth, and membrane filters were used as experimental media in many environmental studies which had environmentally sourced donors or recipients (**Supplementary Table S1**).

**Figure 2:**
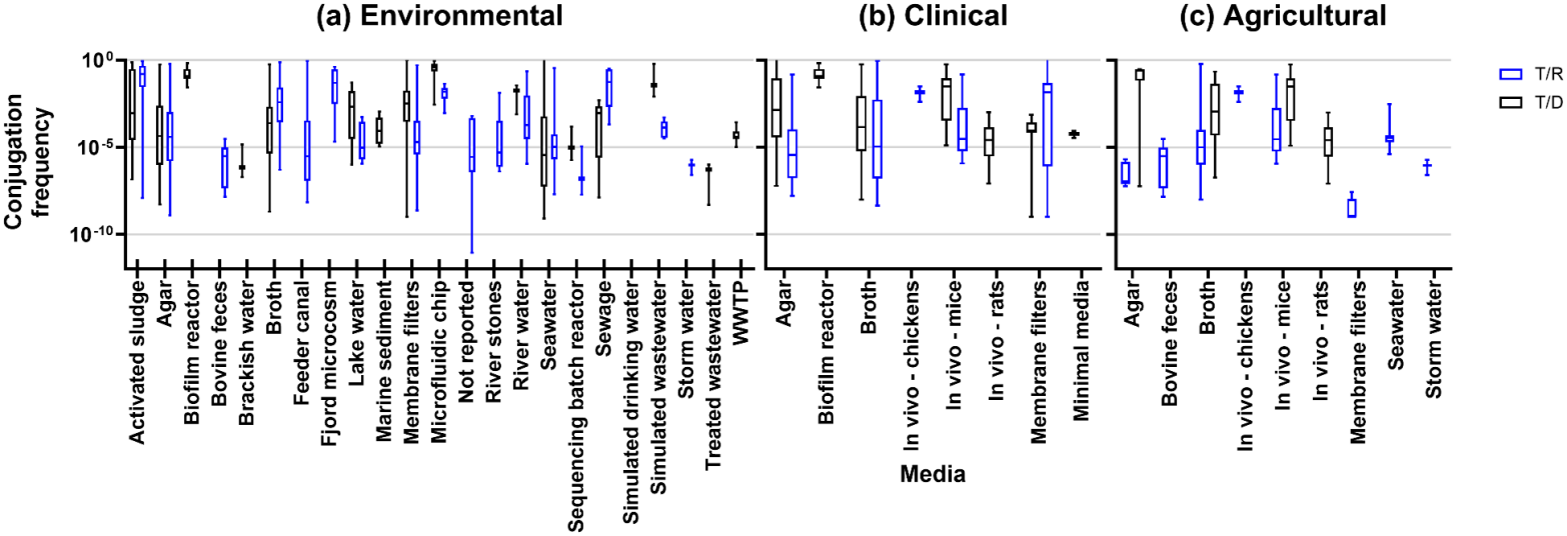
Conjugation frequencies by experimental media type for **(A)** environmental studies, **(B)** clinical studies, and **(C)** agricultural studies.

Measurable (nonzero) conjugation frequencies in environmental studies (n=71/113) ranged from 7.9×10^-10^ to 3.6 T/D (n=30/71) and 8.7×10^-12^ to 8.6×10^-1^ T/R (45/71), illustrated in **Figure 2**. Using wastewater as an experimental media yielded rates of 4.9×10^-9^ to 1.0×10^-6^ T/D for treated wastewater (1/71), 5.0×10^-9^to 8.8×10^-4^ T/D (1/71) and 3.0×10^-6^to 1.0×10^-5^ transconjugants/recipients (T/R) for activated sludge (1/71), 3.3×10^-9^ to 4.8×10^-3^ T/D (2/71) and 2.0×10^-4^ to 2.6×10^-4^ T/R (1/71) for raw wastewater, and 7.9×10^-3^to 6.2×10^-1^ T/D for simulated wastewater (1/71) (**Figure 2A**).

#### 3.1.2 Clinical frequencies

Conjugation rates that were measured and quantified in clinical settings was the next largest category in the review (n=42/113). These were categorized based on isolates taken from clinical settings, usually from human patients. Clinical studies predominantly measured conjugation rates in agar (20/42), broth (13/42), and *in vivo* (7/42 [human 1/7, mice 5/7, rat 2/7, and chicken 1/7]). Other media include membrane filters (5/42), minimal media (1/42), or a biofilm reactor (1/42) using clinical isolates of donors or recipients. Clinical studies observed conjugation frequencies from 1.0×10^-9^ to 3.25×10^-4^ T/D and 1.00×10^-9^ to 3.16×10^-4^ T/R (**Figure 2B**).

#### 3.1.3 Animal/agricultural frequencies

Compared with clinical and environmental sources, the agricultural environment is also composed of bacteria-rich hotspots for AMR: soils, manure, and wastewater. With this in mind, we assessed the studies for any isolates of animal or food origin or setting, resulting in 16 studies designated as animal/agricultural. These studies had donors or recipients isolated from tannery wastewater (1 study), mice or rats (5 studies), bovine (2 studies), poultry (4 studies), dairy (2 studies), or fish (2 studies). Observed conjugation frequencies were between 5.9×10^-8^ to 0.56×10^-1^ T/D and 1.00×10^-9^ to 6.0×10^-1^ T/R (**Figure 2C**).

### 3.2 Conjugation rates

**Figure 3:**
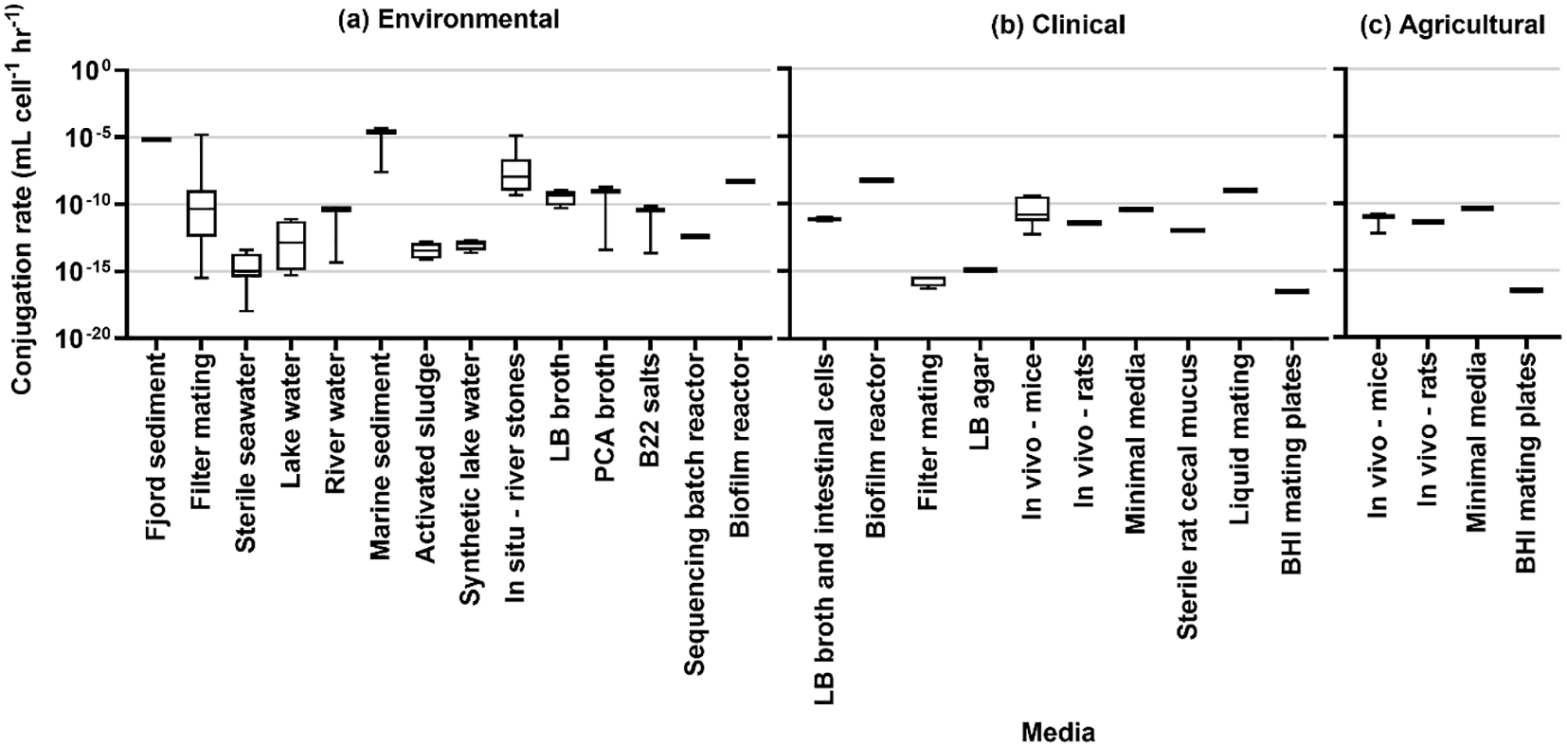
Conjugation rates by experimental media type for **(A)** environmental studies, **(B)** clinical studies, and **(C)** agricultural studies.

**Figure 4:**
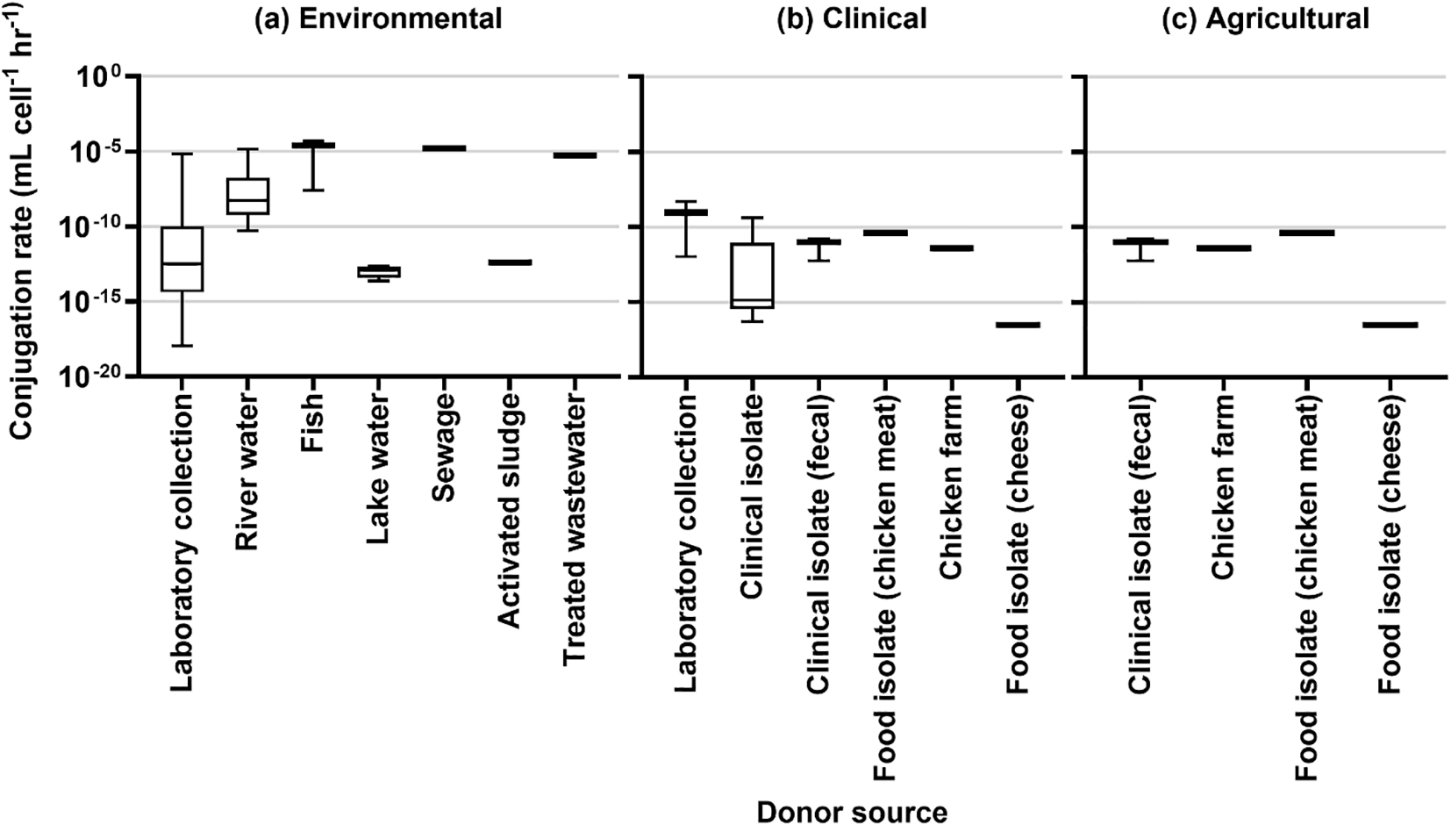
Conjugation rates by source of donor for **(A)** environmental studies, **(B)** clinical studies, and **(C)** agricultural studies.

**Figure 5:**
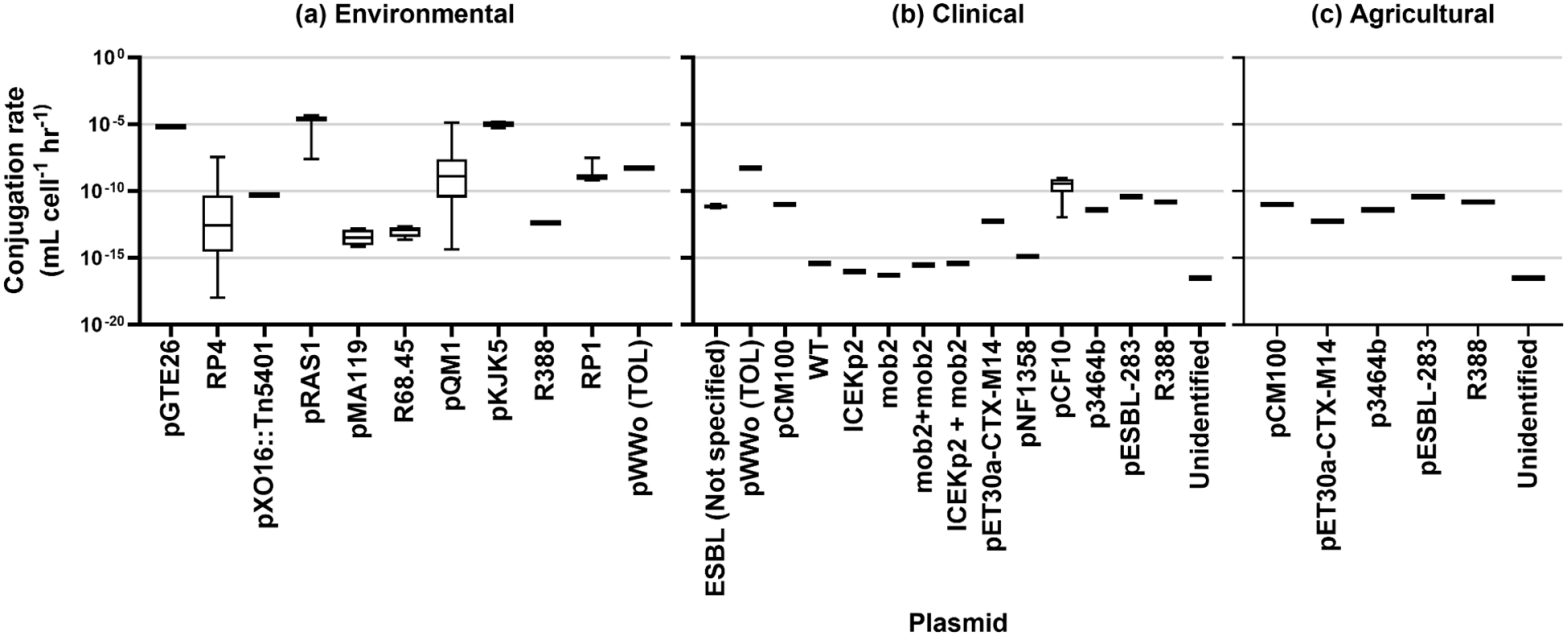
Conjugation rates by plasmid type for **(A)** environmental studies, **(B)** clinical studies, and **(C)** agricultural studies.

The results of rate estimation for a common unit are shown in **Figure 3** based on media type, **Figure 4** by the source of the donor strain, and **Figure 5** by plasmid. The rates of transfer varied over 12 orders of magnitude, ranging from 1.1×10^-18^ to 4.9×10^-5^ for environmental, 5.1×10^-17^ to 5.2×10^-9^ for clinical, and 5.7×10^-13^ to 4.110^-11^ for agricultural studies in units of ml cell^-1^ h^-1^. **Figure 3** clearly highlights the studies with the highest rates were conducted in fjord sediment (Barkay et al., 1995), marine sediment (Sandaa and Enger, 1994), or *in situ* on river stones (Bale et al., 1988). When plotted by donor source, the highest rates were from fish (transferred in marine sediments (Sandaa and Enger, 1994)), sewage (raw or inlet wastewater) and treated wastewater (Jacquiod et al., 2017). For all estimated rates, environmental studies had higher rates (**Figures 3**, **4**). The higher orders of magnitude for environmental studies (up to 10^-5^ ml cell^-1^ h^-1^) resulted in mean rates of 1.26×1010^-6^, 3.18×10^-10^ and 1.2×10^-11^ for environmental, clinical, and agricultural respectively whereas median rates were similar, at 5.9×10^-12^, 4.6×10^-12^, and 6.9×10^-12^, respectively.

## 4 Discussion

Developing quantitative mechanistic models of HGT across different scales has been identified as a key gap for understanding the spread of AMR (Moralez et al., 2021). Quantified conjugation rates can be used as inputs in models to understand mechanisms of the development of AMR, the resulting microbial community population dynamics (Lopatkin et al., 2016b, 2017), and the potential of AMR risks to public health from environmental or other exposures (Njage and Buys, 2017; Schoen et al., 2021). This is especially key as not all HGT events result in meaningful changes in protein structure or function (Arnold et al., 2022). Current literature focused on quantifying HGT has not fully addressed the impact of environmental factors, such as variable cell density, nutrient access, or growth conditions as populations are transported through different media (e.g., wastewater discharged into surface water) (Thomas and Nielsen, 2005). HGT is difficult to measure *in situ* and therefore presents challenges for quantification (Moralez et al., 2021). Furthermore, identifying the host of ARGs in complex matrices is non-trivial (Eramo et al., 2019), relying on techniques such as single cell sorting and whole genome sequencing to understand host-recipient dynamics (Wei et al., 2021). As a result, reliance on bench-scale co-culture experiments is common for quantifying and understanding HGT dynamics.

This study expands on a previous meta-analysis (Sheppard et al., 2020) to provide a dataset of values that could be used in further modeling efforts specific to environmental and public health applications. While the previous study primarily interrogated plasmid-specific variables (e.g., size, type, etc.), we focused on evaluating conjugation experiments and rates representative of environmental or clinical sources and media. Furthermore, the current work provides information from over 100 additional studies, the majority of which were performed under bench-scale conditions.

This review was motivated by the ever-increasing consumption of antibiotics and thus their contamination in the environment (Polianciuc et al., 2020), and the pressures exerted by antibiotics in the environment even at low concentrations (Yim et al., 2006). In addition, hospitals and clinical environments are critical reservoirs and hotspots of AMR and ARB (Edelsberg et al., 2014), with richer ARB communities and increased dissemination of antibiotics when comparing hospital wastewater to municipal wastewater (Hassoun-Kheir et al., 2020). Despite the identified reservoirs for emergence and spread of clinical ARB (Hocquet et al., 2016; Gordon et al., 2017; Weingarten et al., 2018), Lerminiaux and Cameron (2019) note that they continue to be understudied and less understood with regards to conjugation quantification. Finally antibiotics have historically been applied to both plant (McManus et al., 2002) and animal agriculture (Mann et al., 2021) to fight diseases or to promote growth. Meat and egg industries are noted for their high throughput and populations, and subsequent high antibiotic use (Manyi-Loh et al., 2018). It has been made clear that anthropogenic impact is critical in dictating rates of dissemination and conjugation in the environment, influenced further by background bacteria or antibiotics (demonstrated by (Händel et al., 2015) where including antibiotics in the experiment increased rates of conjugation by over 6 orders of magnitude, for example).

One major limitation of this review was the wide variety of data reporting practices for conjugation experiments. This limits the quality of meta-analysis as the ratios of transconjugants, recipients, and donors reported are not consistent, are not typically reported as a function of time, and the donor or recipient identities are missing in many cases. While the experimental timing was reported in most cases (101/113 studies), multiple time points are rarely measured, limiting full characterization of kinetic processes including frequency and directionality of ARG and/or plasmid transfer over time. Lastly, timing of antibiotic administration also plays a role in plasmid transfer rate (Ma et al., 2023), which is typically not accounted for.

The classification scheme used (environmental, clinical, and/or agricultural) was designed to inform comparisons across different matrices. The clinical studies were not necessarily representative of human or other *in vivo* environments, but rather reflective of the media in which the experiment was performed. Nevertheless, these categorizations are useful for informing predictions in different media. Guidelines have been proposed for quantifying conjugation rates and reporting their associated meta-data, including a checklist of meta-data for reporting such as experimental variables, environmental parameters, biological samples, quantification methods, selective conditions, sample preparation, and protocol details (Kosterlitz and Huisman, 2023). The authors specifically define “population ratios” rather than “rates” due to the unit differences and describe other methods for computation of conjugation rates. Additionally, web-based applications have been developed to estimate conjugation rates from experimental data and to account for differences in growth and conjugation rates (Huisman et al., 2022).

For modeling and risk assessment, recent studies have applied frameworks to assess risks related to AMR, but have ignored or made assumptions regarding the impacts of HGT on resistant bacteria populations (Goh et al., 2023; Nahim-Granados et al., 2024; Quon and Jiang, 2024; Schoen et al., 2021). In addition, it remains unclear to what extent conjugation impacts human dose response to pathogenic and antibiotic resistant bacteria (Chandrasekaran and Jiang, 2019), though it is important that proper units (such as Simonsen endpoint estimation demonstrated here) be available for better alignment and inclusion with previously established models for population dynamics (Lopatkin et al., 2016a). Much like established dose response models for quantitative microbial risk assessment, a limitation of *in vivo* conjugation experiments is the use of animal trials for probability estimation due to lack of human data (Haas, 2015). However, the reviewed studies provide a basis for estimation and the impact of intestinal cells or *in vivo* processes found in this review (Faure et al., 2009; Hirt et al., 2018; Maisonneuve et al., 2000) should be further examined for their rates of conjugation related to potential risk models.

Previous studies note the importance of non-antibiotic factors in influencing HGT such as bacteria density, temperature, and nutrients (Jiang et al., 2022; Li and Zhang, 2022). While our study aimed to collect and summarize conjugation rates across literature, it is beyond the scope to estimate the quantitative role of these factors, as estimated rates and frequencies were also dependent on factors such as strain, experimental media, and donor/recipient source. Thus, it is challenging to quantify the extent conjugation rates impact the risk of AMR strain development in environmental matrices and subsequent dissemination to relevant receptors, and should be explored further along.

As a result of the literature review, several research gaps were identified. A clear lack of quantitative measurements of conjugation exists among environments of interest for environmental exposure modeling, including biofilm environments. This, coupled with various, incomplete, and inconsistent reporting conditions, leave many open questions in terms of identifying factors that are most impactful for conjugation rates. For example, biochemical variables can drastically impact microbial physiology, though are rarely included in analyzed studies. Even studies that examine the same environment may have significantly different physicochemical conditions, and thus may not be directly comparable. Standardizing the reporting of environments will be critical moving forward to derive consistent rates for predictive uses. In addition to kinetic conjugation rates, growth rates of one or both of the parental populations are also important parameters to inform the resulting selection dynamics in a given environment. Identifying environmental factors that independently impact the growth can therefore also be useful from a predictive modeling standpoint. Finally, the reporting of conjugation rates as well as matrices shows considerable variation. While some environmental matrices are categorized generally (such as surface waters vs. lakes and rivers more specifically), orders of magnitude differences are noted in conjugation frequencies and rates, and specific factors

Beyond the cellular level, field measurements have indicated that ARG can accumulate within wastewater biofilms (Medina et al., 2020), which could also have implications for wastewater monitoring and wastewater-based epidemiology (Morales Medina et al., 2022). There is a need to map processes occurring at a small-scale in various environments onto processes relevant for human or ecological exposure and/or infection and risk; existing models have concluded that conjugation was not a risk driver in environmental exposure scenarios but could be improved by better characterization of these rates under different conditions (Njage and Buys, 2017; Schoen et al., 2021). Studies covered in this review included the addition of additives (e.g., yogurt, milk, probiotics, etc.) which may not mimic conjugation under realistic conditions that are relevant for understanding modeling caveats and extrapolating to scenarios beyond the specific experimental conditions. Additionally, future reviews could be expanded to include quantitative databases of HGT and other mechanisms relevant to the development of AMR apart from conjugation (e.g., mutation, transformation, and transduction). As integration of computational and experimental approaches is advanced, there is the potential for filling in key research gaps regarding within-host HGT (Sousa et al., 2023).

## Data Availability

The original contributions presented in the study are included in the article/**Supplementary Material**. Further inquiries can be directed to the corresponding author.
