# Supplementary material for: Quantifying conjugation rates in clinical and environmental matrices: a systematic review to inform risk assessment
Source: Front Microbiomes. 2025 Jan 17;3:1490240. doi: 10.3389/frmbi.2024.1490240 (PMC12993507; doi:10.3389/frmbi.2024.1490240)
Supplement: Supplementary file 2 [file Supplementaryfile2.docx]

**Supporting Information for:**

**Quantifying conjugation rates in clinical and environmental matrices: A systematic review to inform risk assessment**

Quon H^a,c^, Ramirez L^a^, Bagwell B^b^, Moralez J^b^, Sheppard, R.J.^d^, Lopatkin AJ^e,f^, Hamilton KA^a,c^*.

^a^ School of Sustainable Engineering and the Built Environment, 660 S College Ave, Tempe AZ 85281

^b^ Department of Biology, Barnard College; New York, NY 10027; USA

^c^ The Biodesign Center for Environmental Health Engineering, 1001 S McAllister Ave, Tempe AZ 85287

^d^ MRC Centre for Global Infectious Disease Analysis & WHO Collaborating Centre for Infectious Disease Modelling, Jameel Institute, School of Public Health, Imperial College London, UK

^e^ Department of Chemical Engineering, University of Rochester, 4510 Wegmans Hall, Rochester NY 14627

^f^ Department of Microbiology and Immunology, University of Rochester, 500 Joseph C. Wilson Blvd, Rochester, NY 14627

*Corresponding author: Kerry A. Hamilton [kerry.hamilton@asu.edu](mailto:kerry.hamilton@asu.edu)

**Supporting Information**

**Methodology**

**Search strategy.** A systematic literature review was performed based on preferred reporting items for systematic reviews and meta-analysis (PRISMA) guidelines(Moher et al., 2015) to identify studies of HGT reported for water, wastewater, and clinical environments. Two databases, Web of Science and PubMed were searched and keywords relevant to conjugation were entered as a string in the search engine. Keywords were searched for water and wastewater: “(conjugative potential OR transconjugants OR conjugation OR horizontal gene transfer OR conjugative gene transfer OR conjugative plasmids OR mobile plasmids) AND (rates OR rate OR efficiency OR dynamics) AND (water OR wastewater OR activated sludge OR biosolids OR drinking water OR reclaimed water OR recycled water OR water reuse OR hospital wastewater OR surface water OR groundwater OR sewage OR stormwater OR sludge OR biosolids OR soil OR crops OR rhizosphere OR food)” or clinical environments “(conjugative potential OR transconjugants OR conjugation OR horizontal gene transfer OR conjugative plasmids) AND (transfer rates OR transfer rate OR efficiency) AND (clinical OR hospital OR human). Titles and abstracts of articles were screened for relevancy by a single reviewer for each section (water and wastewater vs. clinical-related publications). Relevant records were imported into a shared Zotero reference library and de-duplicated prior to review. Full texts were associated for articles deemed to meet inclusion criteria. For relevant reviews identified (Alderliesten et al., 2020; Ashelford et al., 2006; Hunter et al., 2008; Sheppard et al., 2020; Sorensen et al., 2005), forward and reverse citation searching was performed to identify additional relevant records. A PRISMA systematic flow diagram was created to describe the systematic literature review process. This diagram included the number of studies that met inclusion criteria at each screening step.

**Inclusion criteria.** A broad inclusion criteria was defined as a study reporting a horizontal gene transfer rate or ratio for conjugation mating experiments involving strains (donor or recipient), matrices, or other aspects originating from different water/wastewater (water, wastewater and any associated unit processes, drinking water, stormwater, hospital water/wastewater, environmental waters), agricultural (feedlots, manure, animals, etc.), or clinical (materials from patient samples, in experiments designed to mimic the human body or clinical environments, etc.) environments. Studies could be associated with one or more categories to warrant their inclusion. A horizontal gene transfer rate was defined as a metric such as a ratio involving transconjugants, donors, and/or recipients over an experimental duration to show the change in transconjugants after a donor and recipient cell are allowed to mate, laterally transferring genetic material. A transconjugant is a recipient cell that acquired the genetic material from the donor cell. Flexibility in conjugation “rates” was exercised to allow for collection across different types of formats and units (e.g., transconjugants/donors * recipients or normalized per unit time). The search, eligibility, and inclusion process are outlined in **Figure S1**.


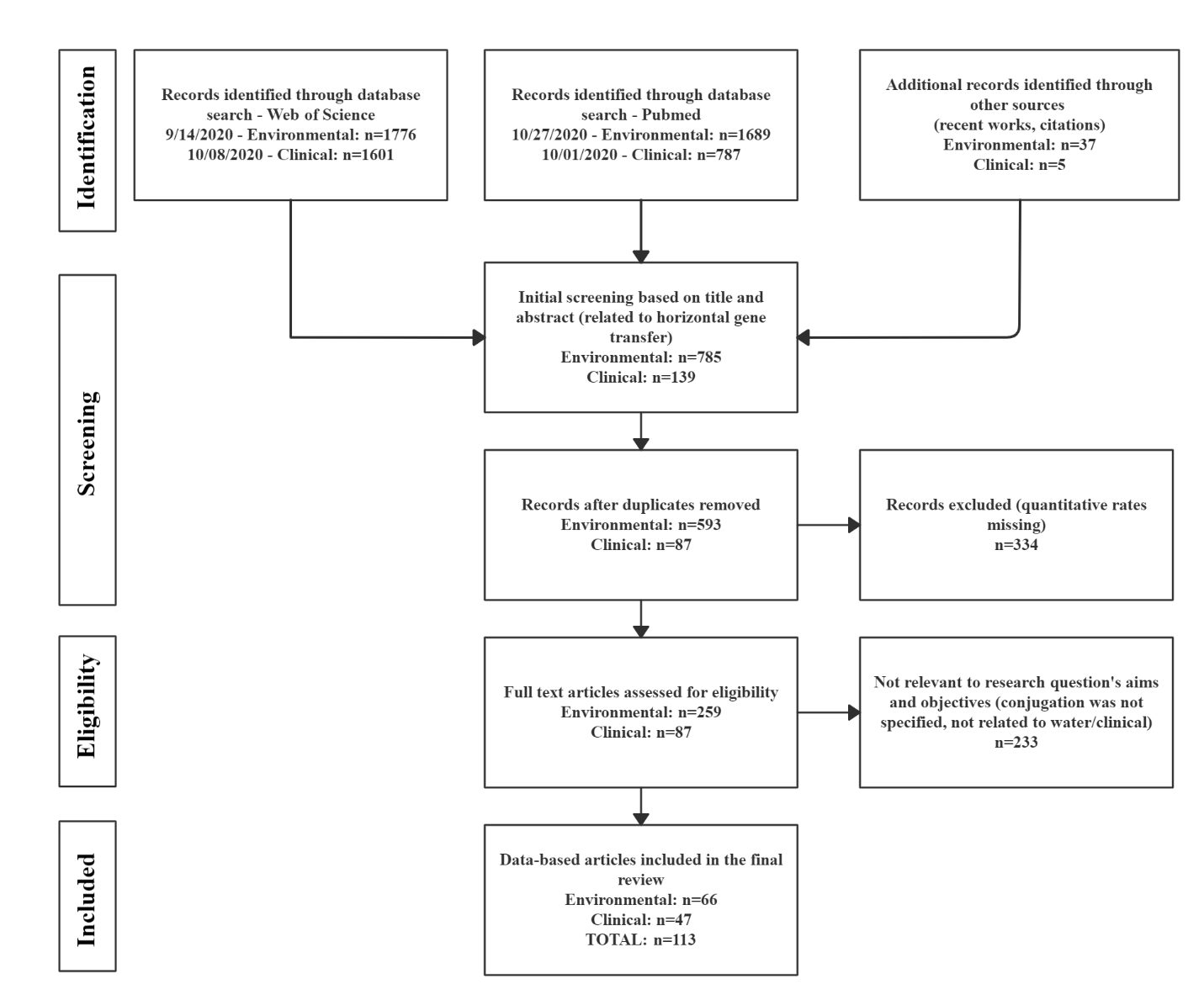


**Figure S1.** Systematic literature review PRISMA process.

**Table S1.** Summary of studies reporting quantitative conjugation rates (E= environmental, C=clinical, A= agricultural; determined based on the experimental media, donor source, or recipient source)

| **^No.^** | **^Ref^** | **^Media^** | **^Donor source^** | **^Donor^** | **^Recipient source^** | **^Recipient^** | **^Resistance transferred^** | **^Units^** | **^Time^** | **^E^** | **^C^** | **^A^** | **^Simonsen^** |
| --- | --- | --- | --- | --- | --- | --- | --- | --- | --- | --- | --- | --- | --- |
| ^1^ | (Alam et al., 2020) | ^Broth, wastewater^ | ^Hospital sewage^ | *^E. coli^*^, Enterobacter^ | ^Hospital sewage^ | *^E. coli^*^, Enterobacter^ | ^Multiple^ | ^T/R^ | ^1 day^ | ^X^ |  |  |  |
| ^2^ | (Alam et al., 2013) | ^Broth^ | ^Hospital sewage^ | ^ESBL^ *^E. coli^* | ^Hospital sewage^ | *^E. coli^* | ^Ampicillin^ | ^T/R^ | ^Overnight^ | ^X^ |  |  |  |
| ^3^ | (Barkay et al., 1995) | ^Fjord microcosm^ | ^Laboratory collection^ | *^P. putida^* | ^Laboratory collection^ | *^P. putida^*^,^ *^P. aeruginosa^*^,^ *^E. coli^*^, Roskilde Fjord indigenous recipients^ | ^Kanamycin^ | ^T/R^ | ^Not reported^ | ^X^ |  |  | ^X^ |
| ^4^ | (Caltagirone et al., 2017) | ^Agar^ | ^Wells, river water, WWTPs^ | *^E. coli^* | ^Laboratory collection^ | *^E. coli^* | ^Cefotaxime, colistin^ | ^T/R^ | ^Not reported^ | ^X^ |  |  |  |
| ^5^ | (Conwell et al., 2017) | ^Agar^ | ^Poultry litter, slurry/soil^ | *^E. faecalis^* | ^Low flow water, septic tank^ | *^Enterococcus faecalis^* | ^Ampicillin, vancomycin, tetracycline, erythromycin^ | ^T/D^ | ^1 day^ | ^X^ |  |  |  |
| ^6^ | (Gekenidis et al., 2020) | ^Filter mating, agar^ | ^Irrigation water^ | *^E. coli^* | ^Laboratory collection^ | *^E. coli^* | ^Kanamycin, rifampin^ | ^T/R^ | ^3 days^ | ^X^ |  |  |  |
| ^7^ | (Eikmeyer et al., 2012) | ^Filter mating, agar^ | ^Municipal WWTP effluent^ | *^E. coli^* | ^Municipal WWTP effluent^ | *^E. coli^* | ^Kanamycin, tetracycline^ | ^T/R^ | ^1-1.04 days^ | ^X^ |  |  |  |
| ^8^ | (Marcinek et al., 1998) | ^Broth, activated sludge^ | ^Hospital sewage^ | *^E. faecalis^* | ^Hospital sewage^ | *^E. faecalis^* | ^Not reported^ | ^T/D^ | ^4 hours^ | ^X^ |  |  |  |
| ^9^ | (Neela et al., 2009) | ^Filter mating^ | ^Sediment, seawater, fish^ | *^Vibrio^* ^spp.^ | ^Laboratory collection^ | *^E. coli^* | ^Tetracycline^ | ^T/R^ | ^1 day^ | ^X^ |  |  |  |
| ^10^ | (Sørensen, 1993) | ^Filter mating, seawater^ | ^Laboratory collection^ | *^E. coli^* | ^Seawater^ | *^P. aeruginosa^*^,^ *^P. fluorescens^*^,^ *^Pseudomonas^* ^spp.,^ *^P. putida^*^,^ *^K. pneumoniae^*^,^ *^K. oxytoca, E. erogenes, C. amalonaticus^* | ^Ampicillin, kanamycin, tetracycline^ | ^T/R, T/D^ | ^Not reported^ | ^X^ |  |  | ^X^ |
| ^11^ | (Sengeløv and Sørensen, 1998) | ^Agar, lake water^ | ^Laboratory collection^ | *^E. coli^*^,^ *^P. fluorescens^*^,^ *^P. putida^* | ^Laboratory collection^ | *^P. putida^*^,^ *^E. coli^* | ^Ampicillin, kanamycin, tetracycline^ | ^T/R^ | ^0.02-2.92 days^ | ^X^ |  |  | ^X^ |
| ^12^ | (Talebi et al., 2008) | ^Filter mating^ | ^Clinical isolates (human), urban sewage, hospital sewage, surface water^ | *^E. faecium^* | ^Laboratory collection^ | *^E. faecalis^* | ^Vancomycin^ | ^T/D^ | ^1-1.04 days^ | ^X^ | ^X^ |  |  |
| ^13^ | (Sugimoto et al., 2017) | ^Agar^ | ^Fish, seawater, wastewater^ | *^P. damselae^*^,^ *^Vibrio^* ^spp., Photobacterium spp.,^ *^Shewanella^* ^spp.,^ *^Proteus^* ^spp.,^ *^Pseudoalteromonas^* ^spp.,^ *^Citrobacter^* ^spp.^ | ^Laboratory collection^ | *^E. coli^* | ^Erythromycin^ | ^T/D^ | ^0.83 days^ | ^X^ |  |  |  |
| ^14^ | (Thomas et al., 2001) | ^River water^ | ^River water^ | *^B. thuringiensis^* | ^River water^ | *^B. thuringiensis^* | ^Tetracycline^ | ^T/D^ | ^7 days^ | ^X^ |  |  | ^X^ |
| ^15^ | (Sørum et al., 2003) | ^Agar, broth^ | ^Fish^ | *^A. salmonicida^* | ^Laboratory collection^ | *^E. coli^* | ^Tetracyclines, trimethoprim, sulfonamides^ | ^T/R^ | ^0.04-2.13 days^ | ^X^ |  |  |  |
| ^16^ | (Verma et al., 2004) | ^Agar^ | ^Treated tannery effluent^ | *^E. coli^* | ^Laboratory collection^ | *^E. coli^* | ^Chromium, copper, zinc, cadmium, nickel, co-trimazole, bacitracin, cephaloridine, ampicillin, polymixin-B^ | ^T/D^ | ^18 hours^ | ^X^ |  | ^X^ |  |
| ^17^ | (Nagachinta and Chen, 2008) | ^Storm water, bovine feces^ | ^Bovine isolates^ | ^STEC^ *^E. coli^* | ^Laboratory collection^ | *^E. coli^* | ^Streptomycin, sulfisoxazole, novobiocin, tetracycline, oxytetracycline^ | ^T/R^ | ^1-13 days^ | ^X^ |  | ^X^ |  |
| ^18^ | (Maher and Taylor, 1993) | ^Broth^ | ^Laboratory collection^ | *^E. coli^* | ^Laboratory collection^ | *^E. coli^* | ^Not reported^ | ^T/R^ | ^0.67-3 days^ |  | ^X^ |  |  |
| ^19^ | (Dong et al., 2019) | ^Membrane filter^ | ^Shrimp^ | *^V. parahaemolyticus^* | ^Fish^ | *^V. campbellii^* | ^Chloramphenicol^ | ^T/R^ | ^0.5 days^ | ^X^ |  | ^X^ |  |
| ^20^ | (Sandaa and Enger, 1994) | ^Marine sediment and seawater^ | ^Laboratory collection^ | *^A. salmonicida^* | ^Laboratory collection^ | *^A. salmonicida^* | ^oxytetracycline, trimethoprim, sulfadiazine^ | ^T/R, T/D^ | ^8 days^ | ^X^ |  |  | ^X^ |
| ^21^ | (Wichmann et al., 2017) | ^Agar^ | ^Laboratory collection^ | *^E. coli^* | ^Laboratory wastewater^ | *^E. coli^*^,^ *^C. freundii, P. fluorescence^*^,^ *^P. putida^*^,^ *^A. hydrophila, B. vesicularis^* | ^Kanamycin^ | ^T/D^ | ^1 day^ | ^X^ |  |  |  |
| ^22^ | (Moura et al., 2007) | ^Agar^ | ^Wastewater treatment: sewage, activated sludge, treated effleunt^ | *^Aeromonas^* ^spp.,^ *^E. coli^* | ^Laboratory collection^ | *^E. coli^* | ^Not reported^ | ^T/R^ | ^Overnight^ | ^X^ |  |  |  |
| ^23^ | (Inoue et al., 2005) | ^Broth^ | ^Laboratory collection^ | *^E. coli^* | ^Activated sludge^ | *^P. putida^* | ^ampicillin, kanamycin, and tetracycline^ | ^T/R^ | ^1 day^ | ^X^ |  |  |  |
| ^24^ | (Geisenberger et al., 1999) | ^Agar, activated sludge^ | ^Not reported^ | *^P. putida^* | ^Activated sludge^ | ^A. delafieldii, A. johnsonii,^ *^A. junii^*^,^ *^P. putida^*^,^ *^Rhizobium^* ^spp.,^ *^S. ficaria, S. liquefaciens, S. capsulata,^* ^indigenous bacteria in activated sludge^ | ^Kanamycin, ampicillin^ | ^T/R^ | ^1 day^ | ^X^ |  |  | ^X^ |
| ^25^ | (Jiao et al., 2017) | ^Agar^ | ^Wastewater effluent and sludge^ | *^E. coli^* | ^Wastewater effluent and sludge^ | *^E. coli^* | ^Not reported^ | ^T/R^ | ^12 hours^ | ^X^ |  |  |  |
| ^26^ | (Dang et al., 2017) | ^Broth^ | ^River water, sediments^ | *^E. coli^* | ^River water, sediments^ | *^E. coli^* | ^Ampicillin, imipenem, gentamicin, erythromycin, tetracycline, sulfamethoxazole, streptomycin,^ | ^T/R^ | ^16 hours^ | ^X^ |  |  |  |
| ^27^ | (Fernandez-Astorga et al., 1992) | ^Broth, sewage^ | ^Sewage^ | *^C. freundii, E. cloacae^*^,^ *^E. coli^* | ^Laboratory collection, sewage^ | *^E. coli^* | ^Ampicillin^ | ^T/D^ | ^2-18 hours^ | ^X^ |  |  |  |
| ^28^ | (Dröge et al., 2000) | ^Activated sludge^ | ^Laboratory collection^ | *^Pseudomonas^* ^spp.,^ *^E. coli^* | ^Laboratory collection^ | *^Pseudomonas^* ^spp.,^ *^E. coli^* | ^Many (See study)^ | ^T/R^ | ^Not reported^ | ^X^ |  |  |  |
| ^29^ | (Walter and Vennes, 1985) | ^Broth^ | ^Wastewater, oxidation ditches^ | *^E. coli^*^, C. freundii^ | ^Laboratory collection^ | *^E. coli^* | ^MAR (Multiple antibiotic resistance)^ | ^T/D^ | ^24 hours^ | ^X^ |  |  |  |
| ^30^ | (Alcaide and Garay, 1984) | ^Broth^ | ^Wastewater, wastewater contaminated surface waters^ | *^Salmonella^* | ^Laboratory collection^ | *^E. coli^* | ^Ampicillin, chloramphenicol, tetracycline, amoxicillin, kanamycin^ | ^T/D^ | ^0.17-0.75 days^ | ^X^ |  |  |  |
| ^31^ | (Ohlsen et al., 2003) | ^Sewage agar, bioreactor with sewage^ | ^Clinical isolates (human)^ | *^S. aureus^* | ^Clinical isolates (human)^ | *^S. aureus^* | ^Gentamicin^ | ^T/R^ | ^Not reported^ | ^X^ | ^X^ |  |  |
| ^32^ | (Laroche-Ajzenberg et al., 2015) | ^Agar^ | ^Surface water, ground water^ | *^E. coli^* | ^Surface water, ground water^ | *^E. coli^* | ^Amoxicillin, ticarcillin, gentamicin, streptomycin, chloramphenicol, tetracycline, clavulanic acid^ | ^T/R^ | ^Overnight^ | ^X^ |  |  |  |
| ^33^ | (Tamanai-Shacoori et al., 1995b) | ^Sewage, brackish water^ | ^Raw wastewater, treated wastewater, brackish water^ | *^E. coli^* | ^Raw wastewater, treated wastewater, brackish water^ | *^E. coli^* | ^Tetracycline, sulfamide, ticarcillin, trimethoprim, streptomycin, chloramphenicol, kanamycin, carbenicilin^ | ^T/D^ | ^0.83 days^ | ^X^ |  |  |  |
| ^34^ | (Jones et al., 1991) | ^Agar, synthetic lake water^ | ^Lake water^ | *^P. aeruginosa^* | ^Not reported^ | *^P. aeruginosa^* | ^Tetracycline, carbenicillin, kanamycin^ | ^T/D, T/DR^ | ^0.75 days^ | ^X^ |  |  | ^X^ |
| ^35^ | (Han et al., 2019) | ^Broth with quaternary ammonium compounds^ | ^Surface water^ | *^E. coli^* | ^Surface water^ | *^E. coli^* | ^Kanamycin, ampicillin, tetracycline^ | ^T/R^ | ^0.67-0.83 days^ | ^X^ |  |  |  |
| ^36^ | (M. J. Bale et al., 1988) | ^Membrane filter, feeder canal^ | ^River water^ | *^P. aeruginosa^*^,^ *^P. putida^*^,^ *^P. fluorescens^*^, P. maltophilia^ | ^River water^ | *^P. aeruginosa^*^,^ *^P. putida^*^,^ *^P. fluorescens^*^,^ *^P. maltophilia, P. cepacia^* | ^Mercury^ | ^T/R^ | ^1 day^ | ^X^ |  |  |  |
| ^37^ | (Tansawai et al., 2019) | ^Broth, agar^ | ^Fecal samples (chicken, duck, human)^ | *^E. coli^* | ^Laboratory collection^ | *^E. coli^* | ^Not reported^ | ^T/D^ | ^Overnight^ | ^X^ | ^X^ | ^X^ |  |
| ^38^ | (Amos et al., 2014) | ^Agar^ | ^River sediment^ | *^C. braakii^*^,^ *^E. coli^*^, C. freundii,^ *^A. media^*^,^ *^K. oxytoca^*^,^ *^P. fluorescens^* | ^River sediment^ | *^E. coli^* | ^Not reported^ | ^T/R^ | ^0.67 days^ | ^X^ |  |  |  |
| ^39^ | (Ozgumus et al., 2007) | ^Broth^ | ^Tap water, spring water^ | *^E. coli^* | ^Tap water, spring water^ | *^E. coli^* | ^ampicillin, tetracycline, or trimethoprim/sulfamethoxazole^ | ^T/D^ | ^Not reported^ | ^X^ |  |  |  |
| ^40^ | (Pickup et al., 1997) | ^Plate mating^ | ^Sewage outfall^ | *^E. cloacae^* | ^Laboratory collection^ | *^E. coli^* | ^Nickel^ | ^T/D^ | ^Not reported^ | ^X^ |  |  |  |
| ^41^ | (M J Bale et al., 1988) | ^Feeder canal^ | ^River water^ | *^P. aeruginosa^* | ^River water^ | *^P. aeruginosa^* | ^Mercury^ | ^T/R^ | ^1 day^ | ^X^ |  |  | ^X^ |
| ^42^ | (McClure et al., 1989) | ^Activated sludge^ | ^Laboratory collection^ | *^P. putida^* | ^Laboratory collection^ | ^Alcaligenes eutrophus,^ *^E. coli^*^,^ *^P. putida^* | ^Kanamycin, tetracycline^ | ^T/D^ | ^0.25-0.33 days^ | ^X^ |  |  |  |
| ^43^ | (Combarro et al., 1992) | ^Broth/selective media, seawater^ | ^Estuary water, laboratory collection^ | *^E. coli^* | ^Laboratory collection^ | *^E. coli^* | ^Ampicillin, tetracycline, chloramphenicol, kanamycin^ | ^T/D^ | ^0.83 days^ | ^X^ |  |  |  |
| ^44^ | (Zhang et al., 2017) | ^Simulated distribution water^ | ^Not reported^ | *^E. coli^* | ^Not reported^ | *^E. coli^*^, S. typhimurium^ | ^Ampicillin, chloromycetin, tetracycline, kanamycin^ | ^Not reported^ | ^0.167-1 day^ | ^X^ |  |  |  |
| ^45^ | (Soda et al., 2008) | ^Activated sludge, broth^ | ^Not reported^ | *^E. coli^*^,^ *^K. pneumoniae^*^,^ *^E. cloacae^* | ^Activated sludge^ | *^E. coli^*^, activated sludge bacteria^ | ^Not reported^ | ^T/R, T/D^ | ^1 day^ | ^X^ |  |  | ^X^ |
| ^46^ | (Breittmayer and Gauthier, 1990) | ^Marine sediment^ | ^Not reported^ | *^E. coli^* | ^Not reported^ | *^E. coli^* | ^amoxicillin, tetracycline, kanamycin^ | ^T/D^ | ^18 hours^ | ^X^ |  |  |  |
| ^47^ | (Bale et al., 1987) | ^Broth, feeder canal^ | ^Laboratory collection^ | *^P. aeruginosa^* | ^Laboratory collection^ | *^P. aeruginosa^* | ^Not reported^ | ^T/R^ | ^1 day^ | ^X^ |  |  | ^X^ |
| ^48^ | (Jacquiod et al., 2017) | ^Membrane filter^ | ^Wastewater treatment plant^ | *^E. coli^* | ^Wastewater treatment plant^ | ^WWTP recipients^ | ^Not reported^ | ^T/DR^ | ^1 day^ | ^X^ |  |  | ^X^ |
| ^49^ | (Qiu et al., 2018) | ^Microfluidic chip^ | ^Laboratory collection^ | *^P. putida^*^,^ *^E. coli^* | ^Activated sludge^ | *^P. putida^*^,^ *^E. coli^* | ^Kanamycin, trimethoprim^ | ^T/R, T/D^ | ^1 day^ | ^X^ |  |  |  |
| ^50^ | (Frank et al., 1996) | ^Sequencing batch reactor^ | ^Activated sludge^ | *^E. coli^* | ^Activated sludge^ | *^E. coli^* | ^Trimethoprim, sulfamide^ | ^T/R, T/D^ | ^1-20 days^ | ^X^ |  |  | ^X^ |
| ^51^ | (Lin et al., 2019) | ^Simulated wastewater^ | ^Laboratory collection^ | *^E. coli^* | ^Activated sludge^ | ^Activated sludge bacteria^ | ^Trimethoprim^ | ^T/D^ | ^2 days^ | ^X^ |  |  |  |
| ^52^ | (Kim et al., 2014) | ^Agar^ | ^Not reported^ | *^E. coli^* | ^Activated sludge^ | *^P. aeruginosa^*^, activated sludge^ | ^amoxicillin, streptomycin, sulfamethoxazole, tetracycline, metallic mercury^ | ^T/R^ | ^0.67 days^ | ^X^ |  |  |  |
| ^53^ | (Goodman et al., 1993) | ^Membrane filter^ | ^Marine strain, laboratory collection^ | *^E. coli^*^,^ *^Vibrio^* ^spp.^ | ^Marine strain^ | *^Vibrio^* ^spp.^ | ^kanamycin, tetracycline, ampicillin^ | ^T/R, T/D^ | ^1 day^ | ^X^ |  |  | ^X^ |
| ^54^ | (Devarajan et al., 2017) | ^Agar^ | ^Surface water sediments^ | *^P. aeruginosa^*^,^ *^P. putida^*^,^ *^P. pseudoalcaligenes^*^,^ *^P. fulva, P. mendocina, P. monteilii, P. moravienis, P. mosselii, P. plecoglossicida, P. stutzeri, P. entomophila^* | ^Not reported^ | *^E. coli^* | ^Not reported^ | ^T/R^ | ^Overnight^ | ^X^ |  |  |  |
| ^55^ | (Roberts et al., 2009) | ^Agar^ | ^Seawater, beach sand^ | *^E. faecalis^*^, S. epidermidis,^ *^E. casseliflavis/gallinarum^* | ^Laboratory collection^ | *^E. faecalis^* | ^Not reported^ | ^T/R^ | ^Not reported^ | ^X^ |  |  |  |
| ^56^ | (Bondarczuk and Piotrowska-Seget, 2019) | ^Broth^ | ^River/lake water and sediment^ | ^Not reported^ | ^River/lake water and sediment^ | *^E. coli^* | ^Not reported^ | ^T/R^ | ^Overnight^ | ^X^ |  |  |  |
| ^57^ | (Li et al., 2019) | ^Microfluidic chip, membrane filters^ | ^Activated sludge^ | *^E. coli^* | ^Activated sludge, wastewater, WWTP effluent^ | *^Pseudomonas^* ^spp., Acinetobacter spp.,^ *^Enterobacter^* ^spp.,^ *^Aeromonas^* ^spp., Clostridia spp., Bacillus spp.,^ *^Bacteroides^* ^spp.,^ *^Klebsiella^* ^spp.,^ *^Ochrobactrum^* ^spp.,^ *^Citrobacter^* ^spp.,^ *^Escherichia^*^-^*^Shigella^* | ^Trimethoprim^ | ^T/D^ | ^1 day^ | ^X^ |  |  |  |
| ^58^ | (Guo et al., 2015) | ^Broth (with saline and wastewater)^ | ^WWTP^ | *^E. coli^* | ^Culture collection (China General Microbiological Culture Collection Center)^ | *^E. coli^* | ^Tetracycline^ | ^T/R^ | ^0.75 days^ | ^X^ |  |  |  |
| ^59^ | (Zhu et al., 2020) | ^Agar^ | ^Surface water^ | *^E. coli^* | ^Not reported^ | *^E. coli^* | ^Colistin^ | ^T/R^ | ^0.67 days^ | ^X^ |  |  |  |
| ^60^ | (Soge et al., 2009) | ^Agar^ | ^Beaches^ | *^S. aureus^*^,^ *^S. capitis, S. vitulinus^* | ^Not reported^ | *^E. faecalis^* | ^Erythromycin, tetracycline^ | ^T/R^ | ^1 day^ | ^X^ |  |  |  |
| ^61^ | (Li et al., 2018) | ^Synthetic wastewater^ | ^Laboratory collection^ | *^E. coli^*^,^ *^P. putida^* | ^Activated sludge^ | ^Activated sludge bacteria^ | ^Trimethoprim, tetracycline, ampicillin, kanamycin, amoxicillin, sulfonamide, streptomycin, mercury^ | ^T/R^ | ^2 days^ | ^X^ |  |  |  |
| ^62^ | (Mach and Grimes, 1982) | ^In situ wastewater treatment, in vitro sterile sewage^ | ^WWTP^ | *^S. enteritidis, P. mirabilis^*^,^ *^E. coli^* | ^WWTP^ | *^E. coli^*^,^ *^S. sonnei^* | ^Sulfadiazine, streptomycin, tetracycline, chloramphenicol^ | ^T/D^ | ^0.125 days^ | ^X^ |  |  |  |
| ^63^ | (O’Morchoe et al., 1988) | ^Lake water^ | ^Laboratory collection^ | *^P. aeruginosa^* | ^Laboratory collection^ | *^P. aeruginosa^* | ^Carbenicillin, tetracycline, kanamycin, mercury^ | ^T/D^ | ^0.083 days^ | ^X^ |  |  |  |
| ^64^ | (Kruse and Sørum, 1994) | ^Broth, seawater^ | ^Bovine milk, minced meat, raw salmon, pig feces, seawater^ | *^E. coli^*^, human pathogenic V. cholerae^ | ^Fish^ | *^A. salmonicida^* | ^ampicillin, tetracycline, streptomycin, trimethoprim, sulfonamides, chloramphenicol^ | ^T/R^ | ^1 day^ | ^X^ |  | ^X^ |  |
| ^65^ | (Dahlberg et al., 1998) | ^Seawater^ | ^Seawater^ | *^P. putida^* | ^Seawater^ | *^Vibrio^* ^strain,^ *^V. fischeri, D. marina^*^,^ *^P. putida^*^, indigenous marine bacteria^ | ^Mercury, kanamycin^ | ^T/R, T/D^ | ^3 days^ | ^X^ |  |  |  |
| ^66^ | (Jutkina et al., 2016) | ^Membrane filters^ | ^WWTP^ | ^Treated effluent bacteria^ | ^Not reported^ | *^E. coli^* | ^sulfamethoxazole^ | ^T/R^ | ^0.014-0.125 days^ | ^X^ |  |  |  |
| ^67^ | (Lyimo et al., 2016) | ^Membrane filters^ | ^Surface water^ | *^E. coli^* | ^Not reported^ | *^E. coli^* | ^Ampicillin, streptomycin, sulfonamide, tetracycline, trimethoprim, ceftazidime, sulfamethoxazole^ | ^T/D^ | ^1 day^ | ^X^ |  |  |  |
| ^68^ | (Malik and Aleem, 2011) | ^Broth^ | ^River water^ | *^Pseudomonas^* ^spp.^ | ^Laboratory collection^ | *^E. coli^* | ^Mercury, copper, zinc, chromium, cadmium, nickel^ | ^T/R^ | ^1 day^ | ^X^ |  |  |  |
| ^69^ | (Shakibaie et al., 2009) | ^Sewage, lake water^ | ^Sewage, lake water^ | *^P. aeruginosa^* | ^Sewage, lake water^ | *^E. coli^* | ^Erythromycin, tetracycline, gentamicin^ | ^T/R^ | ^1 day^ | ^X^ |  |  |  |
| ^70^ | (Mukherjee and Chakraborty, 2007) | ^Broth^ | ^River water^ | *^Escherichia^* ^spp.,^ *^Enterobacter^* ^spp.,^ *^Providencia^* ^spp.,^ *^Salmonella^* ^spp.,^ *^Proteus^* ^spp.,^ *^Citrobacter^* ^spp.,^ *^Kluyvera^* ^spp.,^ *^Serratia^* ^spp.,^ *^Shigella^* ^spp.^ | ^Laboratory collection^ | *^E. coli^* | ^Ampicillin, cefotaxime, cephalexin, tetracycline, kanamycin^ | ^T/D^ | ^0.21 days^ | ^X^ |  |  |  |
| ^71^ | (Zhao et al., 2017) | ^Agar^ | ^Hospital wastewater^ | *^E. coli^* | ^Hospital wastewater^ | *^Actinoplanes^* ^spp.^ | ^Erythromycin^ | ^T/R^ | ^0.33-1.58 days^ | ^X^ | ^X^ |  |  |
| ^72^ | (Machado and Sommer, 2014) | ^Minimal media^ | ^Clinical isolate^ | *^E. coli^* | ^Clinical isolate^ | *^E. coli^* | ^Cefotaxime^ | ^T/D^ | ^.083 days^ |  | ^X^ |  | ^X^ |
| ^73^ | (Hardiman et al., 2016) | ^Broth, membrane filters^ | ^Clinical isolate (patient)^ | *^K. pneumoniae^* | ^Clinical isolate (urine, thigh wound)^ | *^K. pneumoniae^* ^,^ *^E. coli^* | ^Kanamycin, carbapenem, gentamicin^ | ^T/D^ | ^0.83 days^ |  | ^X^ |  |  |
| ^74^ | (Kuo et al., 2018) | ^Agar^ | ^Clinical isolate^ | *^E. faecium^* | ^Not reported^ | *^E. faecium^* | ^Vancomycin^ | ^T*DF/R^ | ^1 day^ |  | ^X^ |  |  |
| ^75^ | (Ma and Bryers, 2013) | ^Biofilm reactor^ | ^Laboratory collection^ | *^P. putida^* | ^Laboratory collection^ | *^P. putida^* | ^Kanamycin^ | ^T/D, T/DR^ | ^1 day^ | ^X^ | ^X^ |  | ^X^ |
| ^76^ | (Fernández-González et al., 2011) | ^Agar^ | ^Not reported^ | *^E. coli^*^,^ *^Bartonella tribocorum^* | ^Not reported^ | *^E. coli, B. henselae^* | ^Kanamycin, ampicillin^ | ^T/D^ | ^0.042 or 0.25 days^ |  | ^X^ |  |  |
| ^77^ | (Yuan et al., 2010) | ^Broth^ | ^Hospital^ | *^B. thuringiensis^*^,^ *^B. anthracis^* | ^Hospital^ | *^B. cereus^*^,^ *^B. thuringiensis^*^, B. anthracis^ | ^Erythromycin^ | ^T/D^ | ^0.21 days^ |  | ^X^ |  |  |
| ^78^ | (Launay et al., 2006) | ^In vivo (mice), membrane filters^ | ^Human fecal sample^ | *^E. lenta, C. symbiosum^*^,^ *^E. faecalis^*^,^ *^E. faecium^* | ^Not reported^ | *^E. faecium^* | ^Fusidic acid, rifampin, vancomycin, tetracycline, spectinomycin^ | ^T/R^ | ^Not reported^ |  | ^X^ | ^X^ | ^X^ |
| ^79^ | (Dmowski and Kern-Zdanowicz, 2020) | ^Agar^ | ^Clinical isolate^ | *^E. coli^* | ^Clinical isolate^ | *^E. coli^*^,^ *^P. putida^*^, C. necator, A. tumefaciencs,^ *^B. subtilis^*^, L. lactis^ | ^Tetracycline, kanamycin, chloramphenicol, spectinomycin^ | ^T/D^ | ^0.083-1 day^ |  | ^X^ |  |  |
| ^80^ | (Hong et al., 2017) | ^Broth^ | ^Clinical isolate^ | *^P. aeruginosa^* | ^Clinical isolate^ | *^P. aeruginosa^* | ^Gentamicin^ | ^T/R^ | ^1 day^ |  | ^X^ |  |  |
| ^81^ | (Pachulec and Van Der Does, 2010) | ^Agar^ | ^Clinical isolate^ | *^N. gonorrhoeae^* | ^Clinical isolate^ | *^N. gonorrhoeae^* | ^Tetracycline, kanamycin, erythromycin^ | ^T/D^ | ^0.21 days^ |  | ^X^ |  |  |
| ^82^ | (Woods et al., 2019) | ^Broth, agar^ | ^Novel strain sExpress^ | *^E. coli^* | ^Not reported^ | ^C. autoethanogenum, C. sporogenes,^ *^C. difficile^* | ^Kanamycin^ | ^CFU mL, T/D^ | ^0.33-3 days^ |  | ^X^ |  |  |
| ^83^ | (Farzand et al., 2019) | ^Agar^ | ^Clinical isolate^ | *^K. pneumoniae^* | ^Clinical isolate^ | *^E. coli^* | ^Not reported^ | ^T/R^ | ^Overnight^ |  | ^X^ |  | ^X^ |
| ^84^ | (Parmeciano Di Noto et al., 2016) | ^Agar^ | ^Clinical isolate^ | *^Shewanella^* ^spp.^ | ^Clinical isolate^ | *^Shewanella^* ^spp.,^ *^E. coli^* | ^Trimethoprim^ | ^T/D^ | ^0.75 days^ |  | ^X^ |  |  |
| ^85^ | (Bertsch et al., 2014) | ^Membrane filters^ | ^Clinical/food isolate^ | *^L. monocyotgenes, L. innocua, L. fleischmannii^* | ^Clinical/food isolate^ | *^L. monocytogenes^*^,^ *^E. faecalis^* | ^Tetracycline + integrase, clindamycin^ | ^T/D^ | ^Overnight^ |  | ^X^ | ^X^ |  |
| ^86^ | (Cho et al., 2014) | ^In vivo- mice^ | ^Clinical isolate^ | ^ESBL^ *^Shigella^* ^sonnei^ | ^In vivo gut microbiome strain^ | *^E. coli^* | ^Cefotaxime^ | ^T/D^ | ^7 days^ |  | ^X^ | ^X^ | ^X^ |
| ^87^ | (Berger et al., 2018) | ^Agar^ | ^Clinical isolate^ | *^E. coli^* | ^Clinical isolate^ | *^E. coli^* | ^Ampicillin^ | ^T/R^ | ^Overnight^ |  | ^X^ |  |  |
| ^88^ | (Peterson et al., 2011) | ^Agar^ | ^Not reported^ | *^Salmonella^* | ^Clinical isolate^ | *^E. coli^* | ^Ampicillin^ | ^T/D^ | ^0.083-1 day^ |  | ^X^ |  | ^X^ |
| ^89^ | (Pearce et al., 1999) | ^Membrane filters^ | ^Not reported^ | *^S. aureus^* | ^Clinical isolate^ | *^S. aureus^* | ^Gentamicin, kanamycin, ethidium bromide, centrimide^ | ^T/R^ | ^0.75 days^ |  | ^X^ |  |  |
| ^90^ | (Zeng et al., 2018) | ^Agar^ | ^Not reported^ | *^E. coli^* | ^Clinical isolate^ | *^C. jejuni^* | ^Kanamycin^ | ^T/R^ | ^0.29 days^ |  | ^X^ |  |  |
| ^91^ | (Varahan et al., 2014) | ^Agar^ | ^Not reported^ | *^E. faecalis^* | ^Clinical isolate^ | *^E. faecalis^* | ^Rifampin, erythromycin^ | ^T/D^ | ^0.167 days^ |  | ^X^ |  |  |
| ^92^ | (Haverkate et al., 2015) | ^In vivo (human)^ | ^Clinical isolate^ | *^K. pneumoniae^*^,^ *^E. coli^* | ^Clinical isolate^ | *^K. pneumoniae^*^,^ *^E. coli^* | ^Meropenem, imipenem^ | ^Transfer events/day^ | ^Not reported^ |  | ^X^ |  |  |
| ^93^ | (Savage et al., 2013) | ^Broth^ | ^Clinical isolate^ | *^S. aureus^* | ^Clinical isolate^ | *^S. aureus^* | ^Gentamicin^ | ^T/D^ | ^0.75-4 days^ |  | ^X^ |  |  |
| ^94^ | (Tong et al., 2014) | ^Agar^ | ^Clinical isolate^ | *^E. coli^* | ^Clinical isolate^ | *^S. aureus^* | ^Herbal concoction^ | ^T/R^ | ^1 day^ |  | ^X^ |  |  |
| ^95^ | (Hirt et al., 2018) | ^In vivo (mice), broth^ | ^Laboratory collection^ | *^E. faecalis^* | ^Clinical isolate^ | *^E. faecalis^* | ^Tetracycline^ | ^T/D^ | ^0.167-7 days^ |  | ^X^ | ^X^ | ^X^ |
| ^96^ | (Netherwood et al., 1999) | ^In vivo (chicken)^ | ^Laboratory collection^ | *^E. faecium^* | ^Not reported^ | *^E. faecium^* | ^Erythromycin^ | ^T/R^ | ^7-48 days^ |  | ^X^ | ^X^ |  |
| ^97^ | (Kirk and Fagan, 2016) | ^Agar^ | ^Not reported^ | *^E. coli^* | ^Clinical isolate^ | *^C. difficile^* | ^Thiamphenicol^ | ^T/R^ | ^0.33-1 day^ |  | ^X^ |  |  |
| ^98^ | (Lécuyer et al., 2018) | ^Agar, broth^ | ^Clinical isolate^ | *^B. subtilis^* | ^Clinical isolate^ | *^B. subtilis^* | ^Kanamycin^ | ^T/R^ | ^0.33-0.83 days^ |  | ^X^ |  |  |
| ^99^ | (Faure et al., 2009) | ^Agar, in vivo (rat)^ | ^Agricultural (chicken farm)^ | *^S. enterica^* | ^Clinical isolate (human)^ | *^E. coli^* | ^Tetracycline^ | ^T/D^ | ^8-31 days^ |  | ^X^ | ^X^ | ^X^ |
| ^100^ | (Bai et al., 2017) | ^Broth^ | ^Clinical isolate^ | *^E. coli^* | ^Clinical isolate^ | *^E. coli^* | ^Cefotaxime^ | ^T/D^ | ^Not reported^ |  | ^X^ |  |  |
| ^101^ | (Carraro et al., 2017) | ^Agar^ | ^Clinical isolate^ | *^E. coli^* | ^Clinical isolate^ | *^E. coli^* | ^Naladixic acid^ | ^T/D^ | ^0.25 day^ |  | ^X^ |  |  |
| ^102^ | (Zeng et al., 2015) | ^Agar^ | ^Laboratory collection^ | *^E. coli^* | ^Clinical isolate^ | *^C. jejuni^* | ^Kanamycin^ | ^T/R^ | ^0.29 days^ |  | ^X^ |  |  |
| ^103^ | (Cafini et al., 2016) | ^Agar^ | ^Clinical isolate^ | *^S. aureus^* | ^Clinical isolate^ | *^S. aureus^* | ^Linezolid^ | ^T/R^ | ^0.67-0.79 days^ |  | ^X^ |  |  |
| ^104^ | (Wang et al., 2013) | ^Broth^ | ^Clinical isolate (lamb/chicken)^ | *^E. coli^* | ^Not reported^ | *^E. coli^* | ^Cefotaxime^ | ^T/D^ | ^Not reported^ |  | ^X^ |  |  |
| ^105^ | (Händel et al., 2015) | ^Minimal media^ | ^Food isolate (chicken meat)^ | *^E. coli^* | ^Laboratory collection^ | *^E. coli^* | ^Amoxicillin, ampicillin^ | ^TC/mL^ | ^0.042-2 days^ |  | ^X^ | ^X^ | ^X^ |
| ^106^ | (Liu et al., 2016) | ^Agar^ | ^Laboratory collection^ | *^E. coli^* | ^Not reported^ | *^R. anatipestifer^* | ^Cefoxitin^ | ^T/R^ | ^0.33-0.83 days^ |  | ^X^ |  |  |
| ^107^ | (Xi et al., 2019) | ^Broth^ | ^Laboratory collection^ | *^E. coli^* | ^Laboratory collection^ | *^P. shigelloides^* | ^Chloramphenicol^ | ^% positive TC among 100 colonies^ | ^2 days^ |  | ^X^ |  |  |
| ^108^ | (Anjum et al., 2018) | ^Membrane filters^ | ^Clinical isolate, food isolate (poultry)^ | *^E. coli^* | ^Laboratory collection^ | *^E. coli^* | ^Cefotaxime^ | ^T/D^ | ^0.021 days^ |  | ^X^ |  |  |
| ^109^ | (Licht et al., 2001) | ^Broth, in vivo (rat)^ | ^Laboratory collection^ | *^E. faecalis^* | ^Laboratory collection^ | *^E. faecalis^* | ^Tetracycline^ | ^mL/cell*hr^ | ^0.083-0.21 days^ |  | ^X^ | ^X^ | ^X^ |
| ^110^ | (Maisonneuve et al., 2000) | ^In vivo (mice)^ | ^Clinical isolate^ | *^E. coli^* | ^Clinical isolate^ | *^E. coli^* | ^Trimethoprim^ | ^T/R^ | ^0.25-60 days^ |  | ^X^ | ^X^ | ^X^ |
| ^111^ | (Licht et al., 2003) | ^Broth^ | ^Clinical isolate (rat)^ | *^E. coli^* | ^Clinical isolate (rat)^ | *^E. coli^* | ^Tetracycline, ampicillin^ | ^TC/total cells^ | ^0.188-1 day^ |  | ^X^ | ^X^ |  |
| ^112^ | (Christensen et al., 2011) | ^Broth^ | ^Clinical isolate, food isolate^ | *^E. faecalis^*^,^ *^E. faecium^* | ^Clinical isolate (bovine), food isolate^ | *^E. faecalis^*^,^ *^E. faecium^* | ^Vancomycin^ | ^T/R^ | ^0.167 days - overnight^ |  | ^X^ | ^X^ |  |
| ^113^ | (Feld et al., 2008) | ^Agar^ | ^Food isolate (dairy)^ | *^L. plantarum^* | ^Laboratory collection^ | *^E. faecalis^* | ^Vancomycin^ | ^T/R^ | ^Overnight^ |  | ^X^ | ^X^ | ^X^ |

**Table S2**. Extracted data for all conjugation studies reviewed (See Appendix File).

**
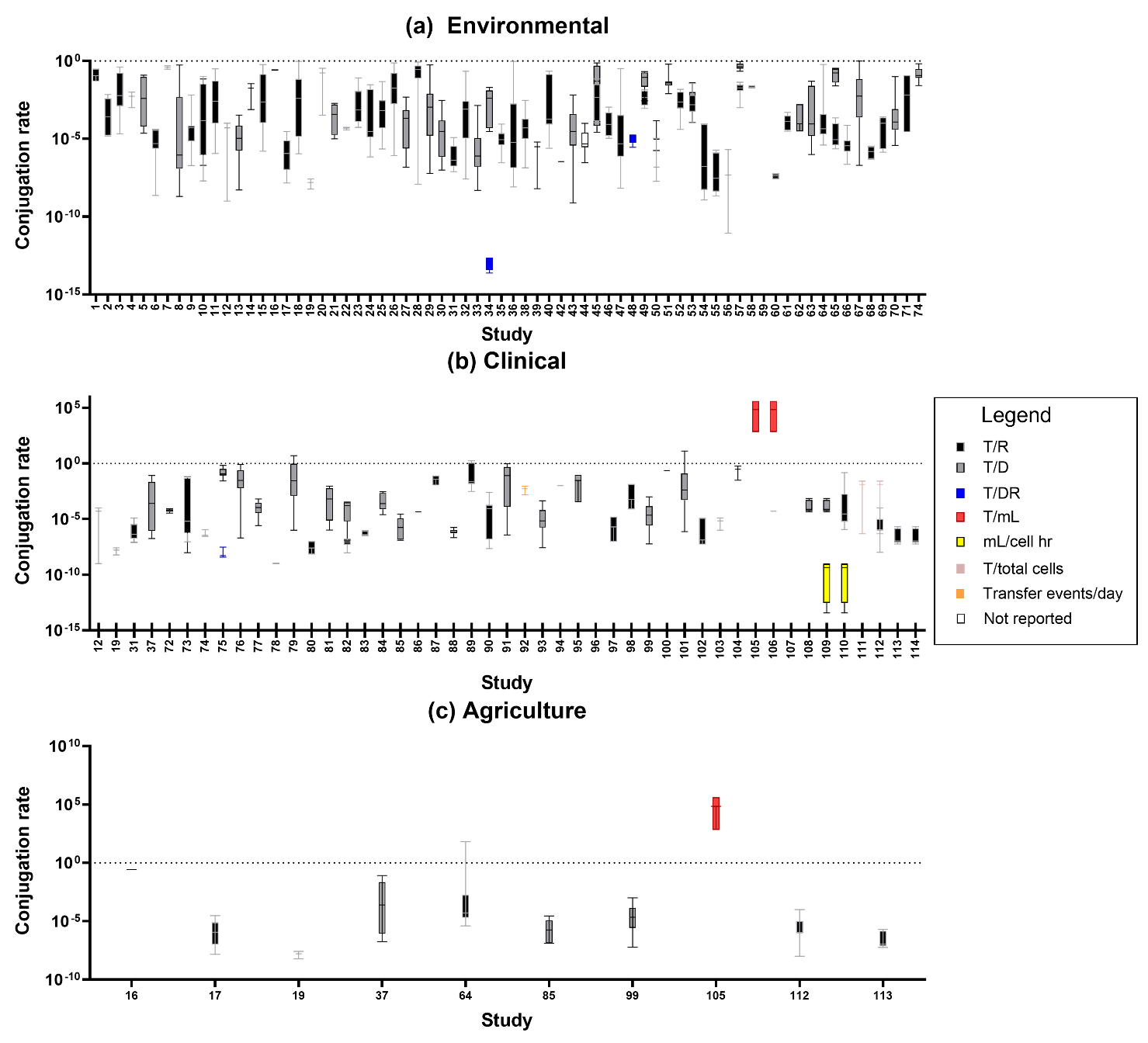
**

**Figure S2.** Conjugation rates and frequencies by study according to the units reported.
